# Updates in Pediatric Drug Allergy

**DOI:** 10.1007/s11882-026-01251-y

**Published:** 2026-02-18

**Authors:** Matthew Robson

**Affiliations:** Division of Allergy and Immunology, Cincinnati Children’s Hospital Medical Center, 3333 Burnet Avenue, MLC 7028, Cincinnati, OH 45229-3026 USA

**Keywords:** Pediatric, Drug allergy, Updates, Antibiotic, NSAID, Beta lactam

## Abstract

**Purpose of review:**

This review is aimed to update clinical allergists on recent international changes in classification, diagnosis, and management of pediatric drug allergy with a focus on antibiotics and NSAID allergy labels.

**Recent findings:**

Guidelines have begun to standardize nomenclature, classification, and diagnostic approaches. In a progressive update, there is now a standard amoxicillin dose for direct provocation challenges, a defined severity scale for immediate reactors, and decreased threshold to drug provocation challenges for patients with serum sickness like reactions. Within antibiotic allergy label investigation, skin testing is being bypassed in favor of direct oral challenges performed in a single day without the use of extended challenges.

**Summary:**

Continued investigation of the index reaction phenotypes, safety of drug challenges, and diagnostic certainty of in vitro studies will continue to advance the field of pediatric drug allergy.

## Introduction

The management of pediatric drug allergy is evolving as the number of studies focused on children grows. The body of literature has begun to support findings previously proven in adults and identify separate, age-appropriate guidance. This review article seeks to bring the clinical allergist up to date on pediatric drug allergy by highlighting changes in international guidelines and the latest publications.

Over the past decade, European and American guidelines for drug allergy have been updated [1]. European guidelines have issued several position papers addressing the management of pediatric drug hypersensitivity reactions. The European Academy of Allergy and Clinical Immunology (EAACI) has published position papers on the diagnosis and management of non-steroidal anti-inflammatory drug (NSAID) hypersensitivity, drug provocation testing in children, updates in nomenclature, and on the evaluation and management of Drug Reaction with Eosinophilia and Systemic Symptoms (DRESS) in pediatric populations [2]. 

In parallel, the American Academy of Allergy, Asthma, and Immunology (AAAAI) updated their drug allergy practice parameters, offering revised recommendations relevant to pediatric patients [1]. Most recently, Accarino et al. have published updated recommendations for drug allergy testing in the United States and Norton et al. have discussed updates in serum sickness like reactions (SSLR) in children [3, 4]. 

This review will discuss updates in pediatric drug allergy and hypersensitivity with a focus on changes in the nomenclature, prevalence, and diagnosis of the three most common classes of medications children are allergic to: beta-lactam antibiotics, non-beta-lactam antibiotics, and NSAIDs [5]. 

## Drug Allergy Nomenclature Updates

Despite taking medications at appropriate doses, children can have adverse drug reactions that are unintended and possibly harmful [6, 7]. Some of these reactions can occur during illness. Infections are an important mechanism to consider in pediatric reactions as viral exanthem can occur independently of medications, but present with benign skin rashes that mimic drug reactions [8]. Additionally, some antibiotics can cause skin eruptions through non-IgE mechanisms including the development of a characteristic rash in patients treated with aminopenicillins during an active Epstein-Barr viral infection [9]. This reaction has not been completely investigated, but is thought to be benign and due to viral induced immune modification causing a temporary reaction [10]. 

On-target adverse drug reactions are caused by the pharmacological activity of the drug and can be predictable [11]. Off-target adverse reactions are unpredictable and are responsible for the minority of reactions [11]. These off-target adverse reactions can be mediated by drug hypersensitivity, typically through antibody or T cell mechanisms, immune receptor activation, or non-immune cell receptor interactions [11]. The 2022 AAAAI Drug Allergy Practice Parameter update emphasized the importance of classifying reactions by time, mechanism, and phenotype [1]. 

Classification of drug reaction timing was previously controversial across continents but has now been standardized [1, 12]. Immediate onset reactions typically occur within one hour, but can occur within 6 h of exposure to the medication [1]. Specific examples of immediate onset drug allergy phenotypes include urticaria and anaphylaxis. Delayed onset reactions occur after 6 h of medication exposure [1]. Delayed onset drug allergy phenotypes include fixed drug eruption, DRESS, and SSLR [1]. 

In addition to phenotypes, the route of administration can influence the timing and severity of allergic responses. Parenteral routes, such as intramuscular and intravenous, are associated with a greater risk of allergic reactions than oral administration [13]. Additionally, reactions have been shown to be more severe in cases of parenteral administration due to faster absorption, shorter latency period to peak concentration, and presentation to the immune system [13]. 

Traditionally, drug hypersensitivity mechanisms have been organized by Gell-Coombs classification with type I (IgE mediated) and type IV (T-cell mediated) being the two most commonly identified in clinical practice [11]. With the identification of new mechanisms of drug hypersensitivity, new classifications have been proposed. Recently, an EAACI position paper put forth nomenclature updates to describe hypersensitivity reactions [14]. Notably, the classification was expanded from types I-IV hypersensitivities to include types V, VI, and VII hypersensitivity reactions. Type VII is the most relevant to this review article as it defines a novel mechanism for drug allergy. Type VII hypersensitivities are defined as an allergic reaction directly triggered by chemical stimulation [14]. Type VII reactions includes cross tolerant NSAID hypersensitivities, non-IgE mediated activation of mast cells through receptors, and the activation of mast cells through complement mediators. NSAIDs-exacerbated respiratory disease, NSAIDs-exacerbated cutaneous disease, and NSAIDs-acute urticaria/angioedema are all now classified as Type VII reactions [14, 15]. Additionally, the activation of mast cells directly through chemical substances are classified as Type VII reactions [14]. 

Novel research has shown mast cells can be activated through non-IgE mediated pathways via the mas-related G protein coupled receptor-X2 (MRGPRX2) expressed on mast cells in the skin [11]. MRGPRX2-associated reactions are a notable example of type VII mechanism [14]. MRGPRX2 is thought to be activated by medications such as icatibant, vancomycin, morphine, ciprofloxacin, and levofloxacin and explains diagnoses such as flushing and immediate urticaria with infusion in the setting of negative skin prick testing [16]. Furthermore, mast cells can be activated through other mechanisms including complement components [17]. Greater consideration is being given to role of chronic diagnoses such as chronic spontaneous urticaria in patients with drug allergy labels [18]. 

New mechanisms of nanoparticle-induced complement activation and cytokine release reactions have been identified and classified as type VII [14]. These mechanisms are clinically relevant because they can mimic classic IgE-mediated anaphylaxis but do not involve adaptive immune sensitization, complicating diagnosis and management [14]. 

Finally, there has been a recent proposed change in nomenclature for the SSLR phenotype [3]. SSLRs are distinct from classic serum sickness due to their clinical presentation and unpredictability [19]. Recent updates showed that despite asymptomatic challenges, the reaction can recur [19]. They are not type I or type III hypersensitivities and laboratory evidence of immune complex deposition is usually absent [3]. This is supported by systematic reviews examining SSLR in large cohorts [20]. While their phenotypical features of rash, joint pain, and fever are similar to true serum sickness reactions, Norton et al. suggest that new terminology would help clarify our understanding of the disease and harmonize future efforts to research the allergy. The authors suggest classifying the clinical presentation of SSLR under the umbrella term of acute urticarial disorders (AUD). Instead of labeling patients as SSLR, highlight features such as AUD-typical when hives are migratory versus AUD-fixed. Providers can add additional qualifiers such as bruising, angioedema, or joint involvement [3]. 

### Drug Allergy Prevalence Update

Adverse drug reactions are common in children and are concerning to parents, often prompting outpatient appointments, emergency visits, and inpatient admissions [21, 22]. A recent review showed that 1.46% of outpatient appointments, 2.09% of emergency visits, and 9.53% of inpatient admissions were due to drug reactions in children [23]. 

When adverse drug reactions occur, they may be inappropriately labeled as drug allergy by parents or providers [24]. Self-reported drug allergies in children ranges from 2.9% to 16.8% internationally [25]. The patients with a reported drug allergy share risk factors of: infantile age, male gender, receiving > 4 medications, and undergoing general anesthesia [23]. However, the majority of patients are later found to be non-allergic when investigated by an allergist [7]. 

Without formal drug evaluation by allergists, many pediatric drug allergy labels persist into adulthood [24]. Reviews have shown that drug allergy labels, particularly in antibiotics, are not inconsequential and lead to broader spectrum antibiotic use which contributes to increased adverse effects, longer hospital stays, and increased rates of infections with *Clostridium difficile*, methicillin-resistant *Staphylococcus aureus*, and vancomycin-resistant *Enterococcus* [26]. 

## Diagnostic Evaluation Updates

### History Gathering

The goals of interviewing a patient with a drug allergy label have remained unchanged. The focus of the history should be on identifying the symptoms of the index reaction, their timing in relation to the suspected medication, all medications given during and directly preceding the reaction, the route of the medication given, and if any medications in the same class have been taken since the index reaction [5]. In the adult population, screening tools for beta lactam allergy, such as PEN-FAST, can help guide specific questions during the interview and help risk stratify the patients drug allergy label [27]. Currently, there are no specific characteristics of the index reaction that can predict allergic outcome to a drug challenge. The PEN-FAST decision tool was not validated in children, and should not be applied to children less than twelve [28]. However, there is ongoing research to examine the phenotype of index reactions to develop other predictive models that are specific for pediatric patients in order to guide risk of allergic challenge outcomes [29]. At present, the provider should establish timing as immediate or delayed, phenotype the drug reaction, and determine the offending agent in order to guide testing.

### In Vitro Studies in Pediatric Drug Allergy

In children, skin testing and drug challenges can be challenging, painful, or contraindicated in the case of severe cutaneous adverse reactions (SCAR) [2, 30]. This has prompted investigation of in vitro modalities such as the basophil activation test and lymphocyte transformation test [30]. The basophil activation test measures CD63 and CD203c on basophils after activation by an allergen. Its use is limited to research of immediate reactions [5]. The lymphocyte transformation test has been used to examine several cases of DRESS, and some studies suggest it to be more sensitive than skin testing for some medications [2]. However, in the United States, there are no widely available, well-validated in vitro tests for drug allergy for pediatric patients that are clinically useful for routine diagnosis [31]. In special circumstances, genetic testing remains important prior to starting abacavir, allopurinol, and carbamazepine due to the associated hypersensitivities [5]. 

### Skin Prick, Intradermal, and Patch Testing in Drug Allergy

Allergy skin testing includes skin prick, intradermal, and patch testing. Currently, skin prick and intradermal testing remain important, safe, and useful tools for clarifying immediate, IgE mediated reactions [5]. A positive skin test is now defined as a wheal at least 3 mm larger than the negative control (with a minimum flare of at least 5 mm) [1]. Patch tests have been used in delayed reactions such as SCARs [5]. The sensitivity of patch testing depends on the medication being tested and the specificity of these tests remains unknown [2]. 

### Direct Provocation Challenges in Pediatric Drug Allergy

Direct provocation challenge remains the gold standard for examining many drug allergy labels. Drug provocation challenges are administered in both inpatient and outpatient settings. Patients are closely monitored for tolerance or developing symptoms. However, there are pediatric specific factors to consider when administering the medication and monitoring for reactions. Drug challenges can be performed on all ages of patients as they have been shown to be safe in infants [4, 32]. 

When choosing a medication for challenge, it is currently recommended to replicate the route of administration and utilize the same product that caused the index reaction, if possible [33]. For some patients, this requires subcutaneous, intramuscular, or intravenous administration. Parenteral challenges require an interdisciplinary approach in monitored conditions, and include involvement with teams such as hospital pharmacy, nursing, allergists, and pediatricians [34]. In specialty centers performing intravenous drug challenges, providers have found smaller doses are required to reach threshold concentrations to elicit reactions, and symptoms are easier to control [33]. The World Allergy Organization released a committee statement outlining the standards for intravenous drug allergy desensitization and delabeling with a special section for pediatric patients [35]. 

When performing oral challenges in children, consider the highest concentration possible in order to minimize volume needed to administer in order to complete the challenge. Additionally, it is appropriate to use flavor masking with other agents when performing oral challenges, as is done in food allergy, through the use of carriers like apple sauce or pudding [4]. 

In order to standardize reaction documentation, there has been an updated release of the USDAR-Peds immediate reaction severity grading scale [4]. Reflecting progress made in the adult population, the USDAR-Peds grading scale has been modified for children and contains a grade for mucocutaneous, respiratory, and cardiovascular features. These are graded from severity, starting with Grade 0 (least severe)and escalating to Grade 4 (most severe), with a separate grading classification for no reaction [4]. Providers performing challenges are encouraged to document using these severity scales in order to increase standardization across published cohorts.

## Antibiotic Allergy Update Overview

Antibiotics have the largest body of literature regarding diagnostic testing because they are frequently reported as the most common drug allergy [36]. The most commonly prescribed class of medications to children internationally are beta lactam antibiotics as they are safe and effective for outpatient and inpatient pediatric infections [37]. Beta lactams are the most common drug allergy label [36]. The prevalence of amoxicillin use has decreased from the 20th century, but the rate of reported allergy remains high due to mislabeling and the use of unverified electronic medical records [11, 38]. 

Diagnostic testing choices for investigation of antibiotic allergy labels are driven by index reaction phenotypes. Low risk phenotypes with a history of beta lactam allergy label, such as benign skin rash can pursue direct oral challenges without skin prick testing. High risk phenotypes like anaphylaxis should pursue skin prick and intradermal testing, and if negative, then direct oral challenge. Recent updates have provided standardization of doses and expanded the definition of low risk phenotypes [4]. 

### Direct Provocation Challenges in Beta Lactam Allergy

Pediatric patients with immediate, benign skin rashes after penicillin class exposure that have no systemic involvement are recommended to pursue a direct provocation challenge without skin prick testing [1]. Benign isolated skin reactions include immediate urticaria and delayed morbilliform rash [1]. Recently, it has been recommended that patients with a history of SSLR pursue a direct provocation challenge to amoxicillin over a single day visit [4]. It is not currently routinely recommended by the AAAAI Drug Allergy Practice Parameters for providers to perform extended challenges following a negative single day challenge as the single day exposure is thought sufficient to capture the majority of delayed reactions [1, 4]. A recent retrospective study in children who performed in hospital and then at home extended challenges, demonstrated positive reactions in 14 children, 3 of which occurred during the at home dose; however, the authors offered alternative explanations to these delayed reactions including whether or not they were due to the in hospital dose or the at home dose [39]. The PROSPECTOR studies are a current randomized controlled trial series investigating whether a single dose is sufficient in clarifying the allergic status of penicillin allergy labeled patients [40]. 

Single day challenges can rule out IgE mediated mechanisms, but Norton et al. have shown that patients with a history of SSLR or AUD can have an unpredictable and episodic course that may return with future therapeutic courses despite a negative challenge [19]. Providers should empower patients who pursue a single day challenge to remove their allergy label but caution them that repeat reactions have been known to occur during future therapeutic courses [19]. 

The recommended dose for penicillin antibiotic challenge in pediatric patients is 250 mg of amoxicillin [4]. This dose was chosen for its safety profile and it has been shown to be sufficient to illicit reactions in all ages and weights for patients with true allergies [4]. It is recommended to administer the dose orally as a single step and observe for sixty minutes. The authors emphasize the efficacy of a single dose but recognize the practicality of a two-step challenge for billing purposes. In the two-step protocol, 50 mg of amoxicillin is administered, followed by fifteen minutes of monitoring, then 200 mg amoxicillin and a final sixty minutes of monitoring, for a total dose of 250 mg of amoxicillin.

Skin prick and intradermal testing for patients with a beta lactam allergy label is now reserved for patients with a history of anaphylaxis as their index reaction. The USDAR-Peds recommends beta-lactam skin prick testing for penicillins, ceftriaxone, and cefazolin when the risk of reaction is high due to a history of anaphylaxis [4]. Skin prick testing for penicillin allergies in the United States should be performed with undiluted benzylpenicilloyl polylysin, penicillin G 10,000 U/mL, and ampicillin 25 mg/mL. Negative skin prick testing can be followed by intradermal testing with the same agents. International centers may have access to a penicilloylpoly lysine minor determinant mixture and sterile amoxicillin [4]. Patients with negative skin testing should undergo drug challenges as previously outlined.

For patients with history of anaphylaxis to ceftriaxone or cefazolin, it is recommended to perform skin prick testing with ceftriaxone 100 mg/mL or cefazolin 330 mg/mL respectively. If negative, providers can pursue intradermal testing at one and ten% of the skin prick testing concentrations. If negative, patients can then undergo two-step challenges. Patients with low risk history can pursue single step intramuscular or intravenous challenges with weight based dosing of 75 mg/kg for ceftriaxone and 30 mg/kg for cefazoline with a maximum dose of 1 gram [4]. All challenges recommend 60 min of monitoring after the final dose has been given. The latest recommendations state that skin prick testing is not recommended for cephalexin or cefdinir of any index reaction (excluding SCAR) and providers can proceed to oral challenge with appropriate weight-based dosing [4]. 

### Direct Provocation Challenges in Non-Beta Lactam Allergy

Non-beta-lactam antibiotics lack validity in skin testing in pediatric patients [41]. The guidelines for diagnosis of these drug hypersensitivity in children follows adult recommendations due to the paucity of available data. Since IgE-mediated reactions are rare in sulfonamides, Accarino et al. recommend pursuing a direct provocation challenge in all non-SCAR index reactions. Furthermore, for patients with drug allergy labels to macrolides, fluoroquinolones, and clindamycin, skin testing is not validated, and these patients should pursue direct provocation challenge [4]. 

## Updates in NSAID Allergy

After antibiotics, nonsteroidal anti-inflammatory drugs (NSAID) are the second most commonly reported allergy [5, 42]. Internationally, ibuprofen is typically the most common NSAID involved [43]. For NSAID allergy assessment, direct oral challenge remains the gold standard [31]. Unlike antibiotic allergies that have a relatively low confirmation rate with direct provocation testing, NSAIDs have a rate as high of 19.6% when undergoing drug provocation testing [43]. This difference is likely due to the underlying pathophysiology of NSAID related reactions resulting from cyclooxygenase inhibition rather than immune mediated mechanisms [42]. 

NSAID hypersensitivity includes both allergic and non-allergic hypersensitivities [42]. Underlying mechanisms of allergy help classify NSAID reactions. Patients are classified as selective or nonselective based on the inciting medication and if it is shared across other members of the same class [44]. Classification of pediatric NSAID hypersensitivity has been further subdivided due to age related patterns. Since younger children most often exhibit non-immunologic reactions and older children have similar reactivity to adults, a division was created at age ten [42]. 

Patients less than ten years of age were either considered to be cross-intolerant reactors or non-cross-intolerant reactors. For children under the age of ten, the previously discussed Type VII reactions of NSAIDs-exacerbated respiratory disease, NSAIDs-exacerbated cutaneous disease, and NSAIDs-acute urticaria/angioedema were grouped into a single classification defined as non-allergic NSAID hypersensitivity. However, they remain separate subsets for children ten to nineteen years old [42]. The other groups of selective NSAID-induced urticaria/angioedema or anaphylaxis and selective NSAID-induced delayed reactions were maintained across all pediatric patients.

While ingestion challenge is recommended, there has not been a universal recommendation regarding dosage and total number of steps [31, 45]. 

## Conclusions

Much like the rest of drug allergy, pediatric drug allergy has entered an era of rapid evolution. Many of the updates seek to standardize the nomenclature and diagnosis of pediatric drug hypersensitivity. There are still areas that require investigation including in vitro testing and selection of challenge doses for medications outside of beta lactam antibiotics. Allergists have an opportunity and responsibility to continue refining evidence-based approaches to delabel harmful allergies and empower families through safe testing to reduce morbidity in children.

## Key References

- Prosty C, Copaescu AM, Gabrielli S, Mule P, Ben-Shoshan M. Pediatric Drug Allergy. Immunol Allergy Clin North Am. 2022;42(2):433-52. doi: 10.1016/j.iac.2022.01.001.
  - ○ This recent, comprehensive review of epidemiology, diagnosis, and management of pediatric drug allergies by medication class and phenotype.
- Accarino JJO, Chow TG, Ramsey A, Rukasin CRF, Gonzalez-Estrada A, Liu AY, et al. A Guide to Pediatric Antibiotic Allergy Testing: A Report From the US Drug Allergy Registry. J Allergy Clin Immunol Pract. 2025;13(5):1018-26.e1. doi: 10.1016/j.jaip.2024.12.036.
  - ○ This impressive, up to date report, while limited in scope to antibiotics, offers progressive and practical recommendations for the clinician that seeks to standardize the field of drug allergy.
- Norton AE, Risma K, Ben-Shoshan M. Serum Sickness-Like Reactions in Children-Is Lifelong Avoidance Indicated? J Allergy Clin Immunol Pract. 2025;13(5):969-77. doi: 10.1016/j.jaip.2025.01.041.
  - ○ This review critically examines serum sickness-like reactions in children and offers updates in nomenclature while questioning the current knowledge of the diagnosis.

## Data Availability

No datasets were generated or analysed during the current study.
